# Myopia Awareness in Australia: Insights from a Nationwide Survey

**DOI:** 10.1007/s44402-026-00151-y

**Published:** 2026-07-29

**Authors:** Mimi Vi, Isabelle Jalbert, Luke Arundel, Alex Huynh, Timothy Li, Pauline Kang

**Affiliations:** 1 School of Optometry and Vision Science, UNSW Sydney, Sydney, New South Wales Australia; 2 Optometry Australia, South Melbourne, Carlton, Victoria Australia

**Keywords:** Awareness, Health literacy, Knowledge, Myopia, Vision

## Abstract

**Background:**

Myopia is an ocular condition that commonly develops in childhood and causes blurred distance vision. It poses a lifetime risk of serious ocular complications that can result in irreversible visual impairment. Early detection and implementation of treatment to slow myopia progression are key to managing the increase in prevalence. This study aims to assess the awareness and understanding related to myopia within the Australian community.

**Methods:**

A cross-sectional nationwide survey was conducted between September and October 2024. The survey was designed using questions drawn from previous myopia awareness studies and tailored for the Australian context. It was administered online and participants were Australian residents, ≥18 years of age. Descriptive statistics and chi-square test of independence were used to evaluate associations between variables of interest.

**Results:**

A total of 1565 responses were analysed, with responses from 68 participants excluded due to their involvement in the eyecare industry. Seventy-nine percent of participants had heard of the term myopia; however, there were aspects relating to risk factors, complications and treatment options that were poorly understood. Only 15% of participants were aware of the existence of treatments to slow myopia progression.

**Conclusion:**

While many participants were aware of myopia, significant gaps existed in understanding its symptoms, risk factors and management. Public health initiatives are needed to improve community knowledge and promote early detection, prevention and treatment to reduce the long-term risk of visual impairment for myopic children.

Key Points

- This study provides evidence that public understanding of myopia is limited and mixed, highlighting gaps in knowledge of myopia treatment and lifetime risk of serious conditions such as retinal detachment.
- The study identifies common misconceptions about myopia, particularly regarding its underlying causes and risks; this limited understanding of the seriousness of myopia may encourage complacency and poor adherence to treatments.
- Public health strategies to raise community understanding of myopia risks and treatment are needed to help support early detection and preventative care.

## Introduction

Myopia or short-sightedness is a refractive error causing distance vision blur. It typically results from accelerated eye growth in childhood, which elongates the eyeball [1]. This excessive eye growth is progressive and irreversible. It increases a child’s lifetime risk of developing vision-threatening complications later in life (e.g., myopic maculopathy, retinal detachment, glaucoma and cataract) [1–3]. Myopia is progressing to become a leading cause of blindness globally if prevalence continues to increase based on predictions by Holden et al. [4]. It is recognised as a disease by the National Academies of Sciences, Engineering and Medicine [5] and included in the International Classification of Diseases (ICD) by the World Health Organization (WHO). Its impact extends beyond visual impairment, affecting academic performance, psychological well-being and overall quality of life. Therefore, it is crucial to prevent myopia progression and minimise its impact [3, 6, 7].

The increasing prevalence of myopia, its related complications and resulting global economic burden have shifted the attitude towards myopia from being a visual inconvenience to recognising myopia as a public health concern and a disease requiring appropriate prevention and management [3–5]. Recent global forecasts estimate that up to 40% of children and adolescents will become myopic by 2050 [8].

In the past 20–30 years, myopia management has evolved drastically with greater acknowledgement and understanding of the associated ocular morbidity [3, 9, 10]. Appropriate care now not only involves risk modifications and correcting myopic visual blur, but also implementing treatment that can reduce progression, thereby reducing the morbidity associated with abnormal eye growth [10, 11]. Although myopia is not reversible, its onset and progression can be mitigated by prescribing effective treatments during early childhood, while the eye is growing [10, 11].

The field of myopia is evolving rapidly, with research on its pathogenesis and treatment continuously emerging that supports or refutes commonly held beliefs about myopia. Consequently, eye healthcare providers must keep their knowledge and practices up to date [12]. Numerous myths and misconceptions surrounding myopia persist within the general community, highlighting the critical role of eyecare providers in advancing community knowledge. Therefore, understanding public awareness and misconceptions is an essential first step towards improving health literacy and supporting timely myopia management. The WHO defines health literacy as the skills required to access, understand and use information and services to make informed health-related decisions to promote and maintain good health and well-being [13]. Health literacy is a social determinant of health, where poor health literacy reduces a patient’s understanding of their condition, treatment and health-associated decision-making, thereby resulting in poorer management [14]. Awareness and understanding of myopia are critical to ensure timely management, improve prognosis and mitigate the adverse consequences of myopia on children’s visual and educational outcomes. Despite this, little is known about community awareness and understanding of myopia, its prevention and management. Gaining this insight will help inform public health strategies and support policy decisions that would enable prevention, early detection and intervention [15]. Thus, this study evaluated awareness and knowledge of myopia as proxies for health literacy within the Australian community.

## Methods

A nationwide cross-sectional community survey was conducted between September and October 2024. The online survey was designed to assess community awareness, understanding and concern around myopia in Australia.

Based on an Australian population of approximately 27 million and utilising a sample size calculator assuming simple random sampling (https://www.qualtrics.com/blog/calculating-sample-size), a minimum of 385 responses was required to achieve a 95% confidence level and a 5% margin of error.

The survey was co-designed with Optometry Australia, the national peak body for optometry. Survey questions were sourced from existing published research on myopia awareness [16–20], modified to suit an Australian context. Additional questions were designed to sample key concerns around awareness and understanding of myopia and common community misconceptions.

Skip logic was used to direct participants to relevant questions based on their responses. This ensured only eligible participants were able to continue the survey and parent- and teacher-specific questions were only displayed to participants who self-reported that they belonged to these cohorts. Survey question responses were of multiple choice, Likert scale, text entry, slider or rank order format.

The survey comprised five sections, including parent-specific and teacher-specific sections, which will be reported elsewhere:

- *Screening and Demographics:* Questions which screened for the inclusion criteria and obtained demographic information (including age, gender, postcode, whether participants were a parent and/or a teacher and if they had any prior experience in the eyecare industry) [19].
- *Awareness and Understanding of Myopia:* Questions designed to gauge participants’ understanding of myopia, including its symptoms, risk factors, potential complications and their level of concern [17–20].
- *Awareness of Myopia Interventions:* Questions to assess participants’ knowledge of strategies and options to reduce the risk of onset and progression of myopia [16].

A copy of all survey questions is provided in Supplementary Table 1.

The survey was administered on the University of New South Wales (UNSW) Qualtrics survey platform (unsw.qualtrics.com) and disseminated nationwide by Empirica Research (empiricaresearch.com.au). Participants were recruited through convenience sampling using a consumer panel at Pureprofile (pureprofile.com). Survey responses were recorded anonymously and did not require follow-up. To minimise selection bias from panel-based convenience sampling, a nationally representative sample was achieved through pre-survey screening questions used to assign participants to pre-determined quota cells for age, gender and Australian states or territories, distributed for a sample target of 1500. This increased target sample enhanced comparability with existing reports, including the 2020 Vision Index [18] and the Australian and New Zealand Child Myopia Report 2022/23 [19].

Participants were selected based on these inclusion criteria:

- Aged 18 years and over.
- Australian resident for at least the past 12 months.
- Proficient in reading and understanding written English, without the need for interpretation or translation services.

As the focus was on assessing community awareness of myopia, individuals who self-reported being involved in the eyecare industry were excluded, and all eligible participants’ responses were included in the analysis irrespective of survey completion

Descriptive statistics and chi-square test of independence were used to evaluate associations between variables of interest. Likert scale responses for ‘Strongly agree’ and ‘Agree’ were collapsed together for some percentage calculations in the discussion of findings. For questions of interest, responses from those who reported having myopia were separated and compared to participants who did not or were unsure.

The findings of this study are reported in accordance with the Strengthening the Reporting of Observational Studies in Epidemiology (STROBE) guidelines [21].

## Results

Responses from 1565 participants were analysed, following exclusion of 68 (4.4%) participants involved in the eyecare industry (i.e., who studied or worked in optical dispensing, optometry, ophthalmology or other aspects). The demographics of survey participants, compared to the Australian Bureau of Statistics (ABS) and Australian Institute of Health and Welfare (AIHW) data sets [22, 23], are summarised in Supplementary Table 2. The gender and state distribution of participants aligned with the general Australian adult population. Comparable self-reported population ethnicity data are not available. A higher proportion of participants reported having a bachelor’s degree or higher, compared with the Australian population. Of the 1478 respondents, 17.4% reported having myopia, 60.8% had no myopia and 21.8% were unsure.

**Fig. 1:**
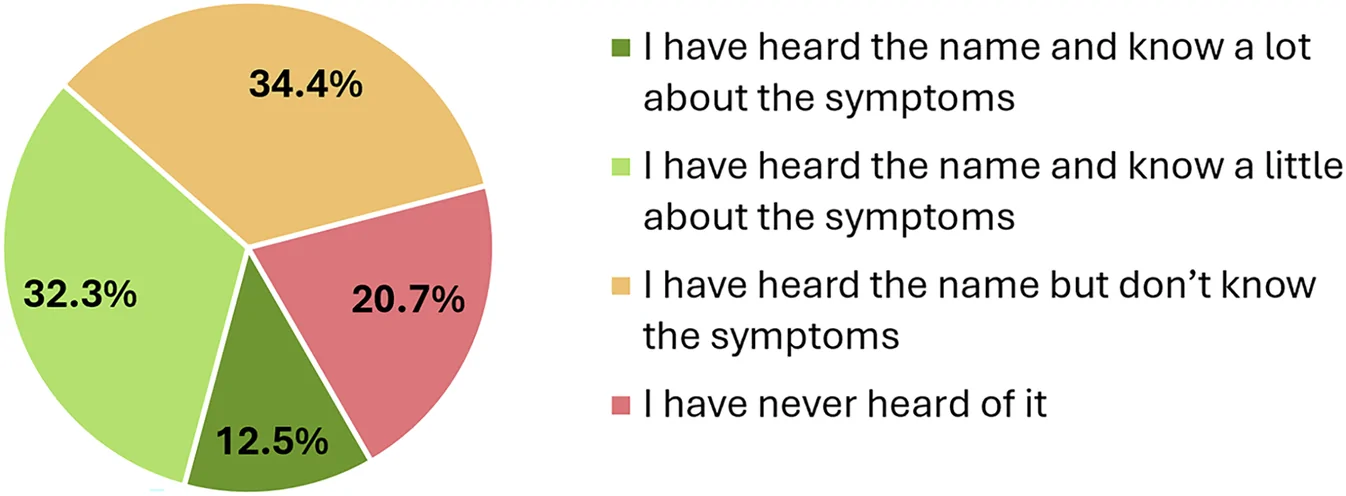
Myopia awareness in Australia based on responses to ‘Select the option that is most true for you regarding the term ‘myopia’ (*n* = 1478).

**Fig. 2:**
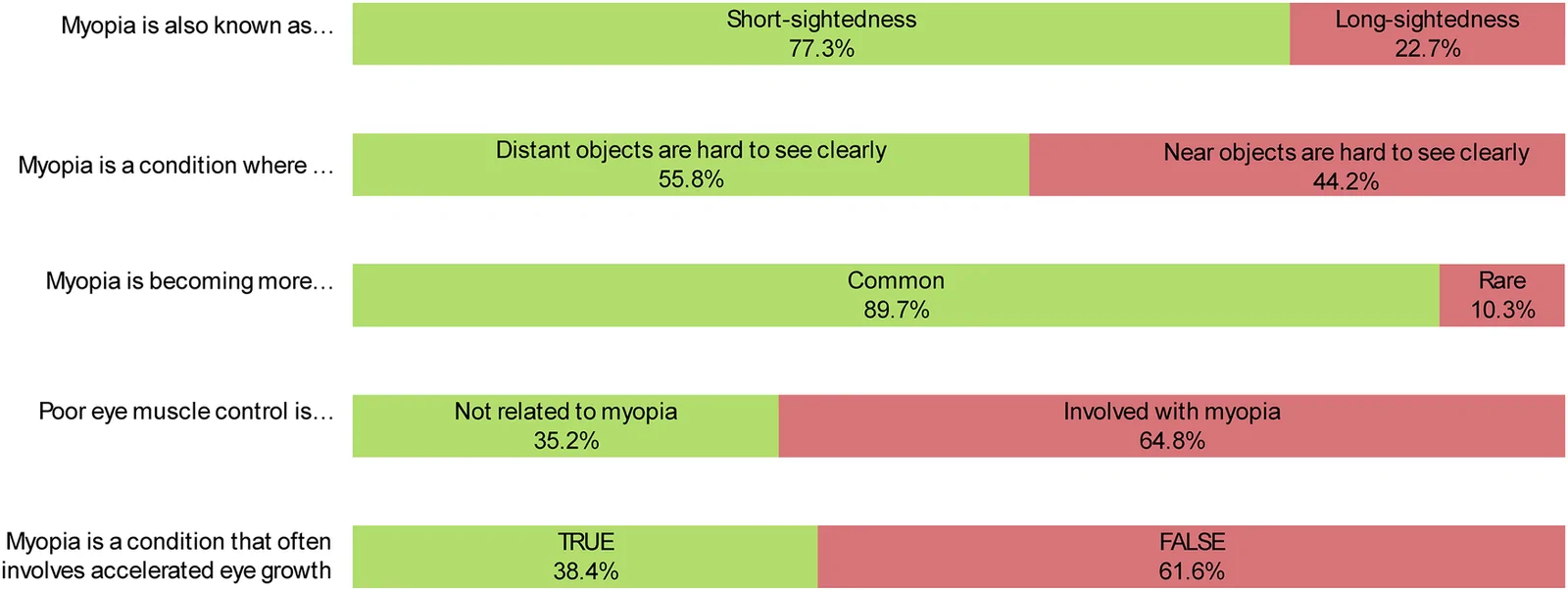
Responses to multiple choice questions which asked participants to select the best option that completes the statement about myopia. Green represents correct answers and red denotes incorrect answers (*n* = 1459).

While most participants (1172; 79.3%) had heard of the term myopia, there was variability in awareness of its symptoms (Fig. 1). Although a similar proportion of participants (1128; 77.3%) correctly associated myopia with the term ‘short-sightedness’, fewer identified it as a condition characterised by distance blur (814; 55.8%) (Fig. 2). A chi-square test of independence showed a statistically significant association between whether participants self-reported having myopia and their understanding that myopia is also known as ‘short-sightedness’, χ^2^ (1, *N* = 1459) = 43.4, *p* < 0.001. Participants with myopia were also more likely to identify the main symptom of myopia correctly, i.e., ‘distant objects are hard to see clearly,’ than those without myopia or unsure of their status (84.4% versus 74.0% and 50.3%, respectively; χ^2^ (1, *N* = 1459) = 103.0, *p* < 0.001). Many participants (946; 64.8%) mistakenly believed that myopia was due to poor eye muscle control rather than accelerated eye growth (560; 38.4%) (Fig. 2). Participants with self-reported myopia showed mixed understanding across different items. While they were less likely to incorrectly associate poor eye muscle control with myopia, χ^2^ (1, *N* = 1459) = 5.3, *p* < 0.02, they were also less likely to identify accelerated eye growth as an underlying cause of myopia χ^2^ (1, *N* = 1459 = 8.2, *p* < 0.004).

**Fig. 3:**
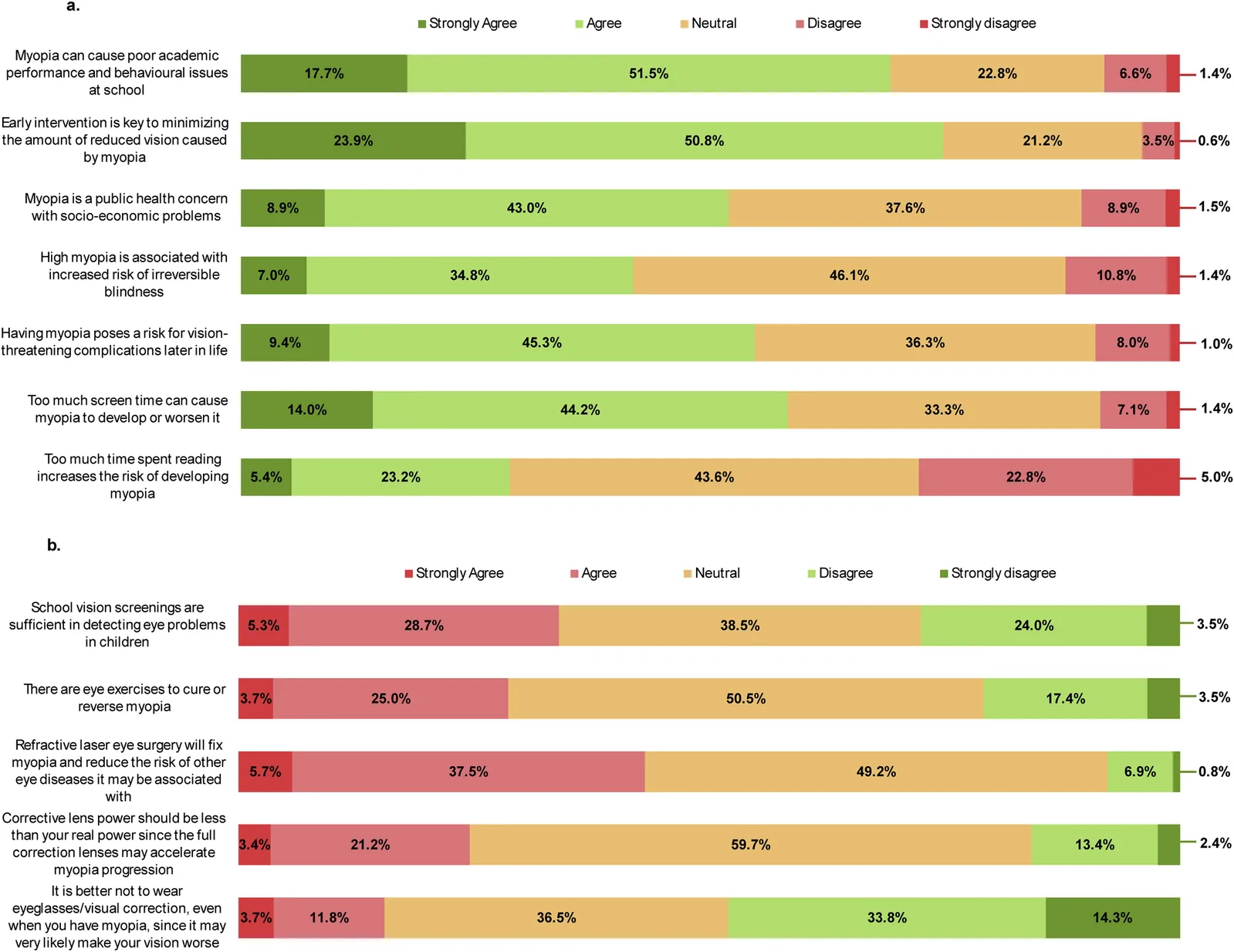
Responses to a Likert scale which asked participants to indicate the extent to which they agree or disagree with (**a**) myopia facts and (**b**) misconceptions. Green indicates the correct answers (*n* = 1446).

When participants responded to statements about myopia using a Likert scale from 1 (strongly disagree) to 5 (strongly agree), there was overall greater agreement to myopia facts (Fig. 3a) and a more neutral position regarding misconceptions about myopia (Fig. 3b). Of the 1437 participants who responded to the question assessing awareness of myopia consequences, more than half (58.7%) selected the response, ‘I have never heard that having myopia can lead to visual health consequences.’ A total of 319 (22.2%) identified cataract development as a complication, 305 (21.2%) identified glaucoma and 264 (18.4%) identified retinal detachment (Supplementary Fig. 1). Overall, there was moderate community concern relating to myopia, with a mean score of 5.6 ± 2.3 out of 10 (10 being very concerned).

**Fig. 4:**
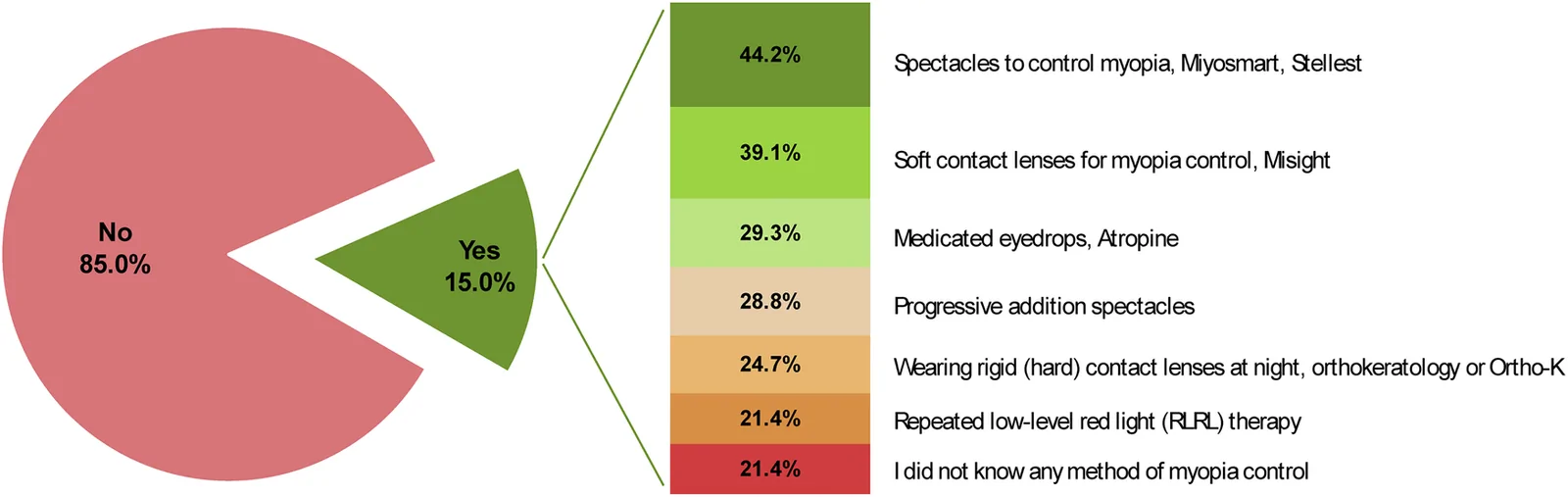
Responses to questions relating to awareness of treatment to slow myopia progression (*n* = 1437) and if aware, the types of methods that participants were familiar with (*n* = 215). Participants could select multiple answers for treatment methods.

Only 215 (15.0%) respondents were aware that treatments to slow myopia progression existed, with myopia control spectacles and soft contact lenses being the most recognised options (Fig. 4). Twenty-one percent of those who were aware of treatments to slow myopia progression were not familiar with specific intervention methods.

## Discussion

Health literacy plays a critical role in disease prevention, including timely diagnosis and appropriate management. Thus, this study aimed to assess myopia health literacy-related gaps in the Australian community using a nationwide online survey. The current national survey measured participants’ awareness of the term ‘myopia’ without the accompanying lay term and found that 1172 (79.3%) of Australians were familiar with the term. This represents an increase in community myopia awareness from the 67.0% reported by Optometry Australia in their 2020 Vision Index report [18]. This finding may partly reflect a higher proportion of self-reported myopes in the study sample (257; 17.4%) compared with the 2020 Vision Index report (7.0%), as individuals living with the condition can reasonably be expected to be more likely to recognise the term. The prevalence of self-reported myopia recorded by the ABS was 28.1% from the National Health Survey (NHS) in 2022 [24]. The exclusion of participants below 18 years of age in the current study may partially explain this discrepancy. Further, the NHS survey used the lay term ‘short-sighted’ instead of ‘myopia’, and this may have facilitated recognition in the general community.

Previous studies have reported low community myopia awareness [25–27]; however, comparisons are difficult because many conflate awareness with understanding of myopia. Awareness refers to the recognition of a health issue while knowledge involves possessing detailed information about the issue [28]. In the current study, myopia awareness was ascertained through respondents’ recognition of the term, whereas knowledge required them to demonstrate an understanding of myopia symptoms, its associated risks and impacts and possible interventions for preventing and managing the condition.

In the current study, participants demonstrated mixed knowledge about myopia, its symptoms and risk factors, with only 185 (12.5%) participants expressing that they knew a lot about myopia symptoms despite 257 (17.4%) participants reporting they had or suspected they had the refractive error. Approximately three in four participants (1128; 77.3%) correctly identified myopia as short-sightedness; however, only about half (814; 55.8%) recognised that myopia is a condition where distant objects are difficult to see clearly. This disconnect shows that recognising the term ‘myopia’ does not guarantee meaningful understanding of the condition.

With regard to understanding myopia pathology, a lower percentage of Australian participants (560; 38.4%) correctly associated myopia with excessive axial elongation compared to a previous survey of Chinese parents (59.2%) [29]. This difference may be attributable to the significantly higher prevalence of myopia in China [29]. Further, 513 (64.8%) participants in the Australian cohort mistakenly believed poor eye muscle control to be involved in myopia pathogenesis, with this misconception occurring at similar rates between those with and without myopia. The mixed understanding revealed here is concerning, as insufficient knowledge may lead individuals to not seek treatment for themselves or their children. This may lead to attitudes that underestimate the detrimental consequences of myopia or inadequately manage the condition [14].

Most participants agreed, to an extent, that myopia is a public health concern and carries a risk of development of vision-threatening complications (Fig. 3a). However, only 604 (41.8%) participants were aware that high myopia can increase the risk of irreversible blindness, while fewer than 20% of participants identified developing retinal detachment as a possible complication of high myopia [2]. In addition, 624 (43.2%) participants mistakenly believed that refractive laser eye surgery cures myopia and lowers the risks of complications such as retinal detachment, consistent with Lan et al.’s finding that 47.0% of Taiwanese patients also held this misconception [30].

Adoption and choice of myopia intervention is heavily influenced by the treating practitioner [31] and by parents’ or carers’ attitude and awareness towards myopia, its consequences and the treatment methods [29]. Despite most participants agreeing that early intervention is key to minimising the consequences of myopia, 1222 (85.0%) were unaware that treatments existed to slow myopia progression. The current study also investigated the prevalence of the belief that wearing a myopic correction can have negative impacts on vision, and found 224 (15.5%) individuals held that misconception to some extent. This percentage was lower than the proportion of participants from a study conducted in Singapore who mistakenly believed that delaying spectacle wear treatment could be of some benefit in the context of myopia management [32]. These common community misunderstandings and misconceptions increase the risk of poor outcomes, as they may lead to complacency, reduced likelihood of seeking routine eye care and delays in myopia diagnosis and treatment [30].

A further barrier to timely myopia treatment is the belief among some practitioners that intervention should begin only after documented progression [9, 10]. This approach is still observed in countries with high myopia prevalence such as Singapore and China [31, 33]. Wolffsohn et al. surveyed eyecare professionals (optometrists, dispensing opticians and ophthalmologists) and reported global variations in myopia management attitudes and strategies. Australasian practitioners had the lowest rates of prescribing single-vision spectacles for progressing young myopes and were among the highest prescribers of soft contact lenses that slowed myopia progression [10]. However, they also expressed the lowest concern about myopia despite their active prescribing. In contrast, concern was greatest among Asian practitioners[10]. Although Australian optometrists generally prescribe appropriately, their relatively low concern, combined with the moderate level of unease seen in the community, suggests the seriousness of myopia and its complications may not be communicated effectively. This highlights the need for strong practitioner training and clear patient communication.

Poor understanding of myopia consequences likely contributes to lower levels of community concern. As with other eye conditions, such as age-related macular degeneration, strong patient education may reduce complacency and support adherence to treatment [34].

Myopia management also involves the provision of advice regarding modifiable environmental risk factors such as increasing time spent outdoors and recommending increased distances for near work [35]. Acknowledging the need for more consistent evidence relating to near work and myopia, current evidence suggests near work distances <30 cm to be detrimental for myopia, and greater than 40 cm to be safe [36, 37]. Only about one in five participants (296; 20.5%) from the current survey identified 40 cm as the safe minimum working distance for screens. Even more concerning, 593 (41.0%) respondents incorrectly considered distances ≤30 cm to be safe. This is more than double the proportion of Singaporeans responding in a similar manner (18.1%) [38].

A key strength of the study was alignment of the participant pool to recent ABS data regarding gender, age and geographical distribution, confirming the survey results as representative of the general Australian community. Those involved in the eyecare industry were overrepresented in the study sample (68; 4.4%) compared with reported national government estimates of 0.5% [39], and their removal from the analysis ensured generalisability of reported results. A primary limitation of the study lies in the self-reported myopia status of participants and potential for misclassification. There is possible education bias with the study sample containing a higher proportion of university-educated participants compared to national data. Future study designs should consider using mixed methods designs to help uncover misunderstandings that surveys alone may miss.

## Conclusion

Despite global increases in myopia prevalence, community awareness and understanding of the condition remains generally mixed in Australia [32, 38]. Nearly 80% of participants in the current study were aware of myopia and reaffirmed the primary role of optometrists in patient education; however, many demonstrated concerning gaps in their understanding of myopia risk factors, its treatments and the potentially severe lifelong implications associated with a myopia diagnosis. Given the narrow window for effective treatment during childhood, the achievement of positive outcomes is highly dependent on ensuring adequate community awareness and understanding. Public health campaigns may include school-based education, social media outreach and practitioner-led counselling that focus on improving understanding of myopia’s causes, symptoms, risk factors and its progressive, irreversible nature.

## Supplementary information

Supplementary Material

## Data Availability

The data that support this study will be shared upon reasonable request to the corresponding author.
