# Supplementary material for: Myopia Awareness in Australia: Insights from a Nationwide Survey
Source: Ophthalmic Physiol Opt. 2026 Jul 29;46(5):1088–94. doi: 10.1007/s44402-026-00151-y (PMC13545111; doi:10.1007/s44402-026-00151-y)
Supplement: Supplementary file 1 [file 44402_2026_151_MOESM1_ESM.pdf]

Supplementary Material

TITLE: Myopia awareness in Australia: Insights from a nationwide survey

JOURNAL NAME: Ophthalmic and Physiological Optics

AUTHORS: Mimi Vi[1], Isabelle Jalbert[1], Luke Arundel[2], Alex Huynh[1], Timothy Li[1] and Pauline Kang[1,*]

AFFILIATIONS:

[1]School of Optometry and Vision Science, UNSW Sydney, Australia, 2052

[2]Optometry Australia, South Melbourne, VIC 3205, Australia

*Corresponding author: p.kang@unsw.edu.au

Supplementary figure legends

Supplementary Fig. 1 Responses to multiple choice questions which asked participants to select all options that they believe to be true regarding consequences of high myopia (n=1437)

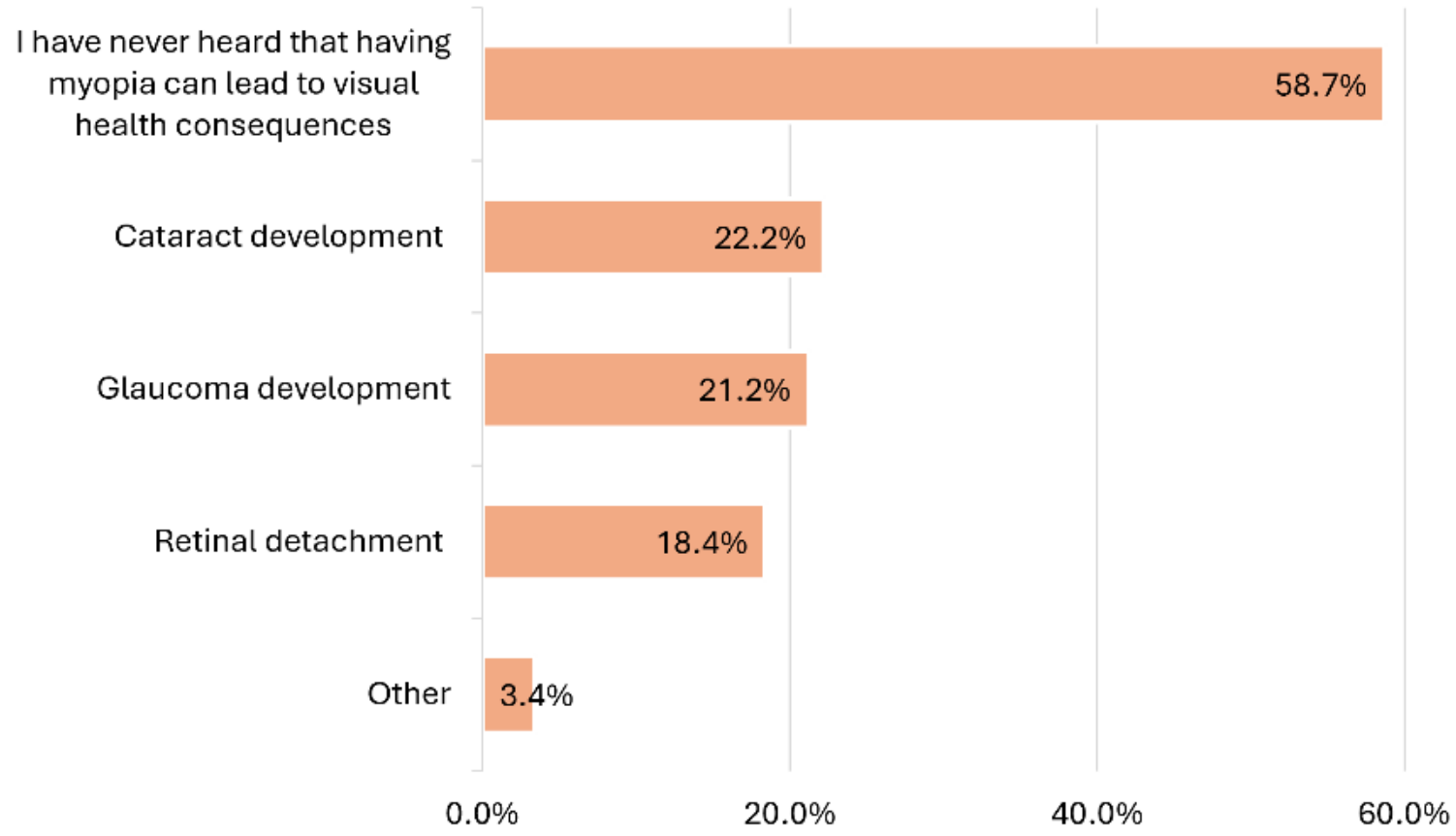

Supplementary tables

Supplementary Table. 1 *Where applicable, correct responses and myopia facts are marked* ***green*** *here for context.*

Myopia Awareness Survey Questions		
	**Question**	**Answer options**
	**Screening Section**	
Q1	Are you proficient in the English language without the need for translation/interpretation services?	• No<br>• Yes
Q2	Have you been an Australian Resident for at least the past 12 months?	• No<br>• Yes
Q3	What is your age?	[Open answer]
Q4	What was your sex recorded at birth?	• Female<br>• Male<br>• Another term (please specify)
Q5	What is your postcode?	[Open answer]
	**Demographics Section**	
Q6	Are you currently or have you previously practised or studied in the eyecare profession? E.g. as an optometrist, ophthalmologist, orthoptist, optical dispenser etc.)?	• Yes<br>• No

Q7	Irrespective of which country you were born in, which of the following best reflects your ethnicity?	• Oceanian (e.g. Australian, Aboriginal and Torres Strait Islander, New Zealand, Maori, Melanesian, Microneasian, Polynesian)<br>• North-West European (British, Irish, Scottish, Welsh, Western European, Northern European)<br>• Southern and Eastern European (Southern European, Southeastern European, Eastern European<br>• North African and Middle Eastern (Arab, Jewish, Peoples of the Sudan, other North African and Middle Eastern)<br>• North-East Asian (Chinese, Taiwanese, Japanese, Korean, Mongolian, Tibetan, Other North-East, Asian)<br>• South-East Asian (Mainland and Maritime South-East Asian including Thai, Vietnamese, Burmese, Filipino, Indonesian, Malay, Timorese, Singaporean etc.)<br>• Southern and Central Asian (Indian, Bengali, Nepalese, Pakistani, Punjabi, Sikh, Sinhalese, Bangladeshi, Sri Lankan, Tamil, Afghan, Armenian, Georgian, Kazakh, Uzbek, etc.)<br>• Peoples of the Americas (North American, South American, Central American, Caribbean Islander)<br>• Sub-Saharan African (Central and West African, Southern and East African)<br>• I'd rather not say
Q8	What is the highest level of qualification or school you have completed?	• Some high school<br>• Completed high school

		• TAFE/trade certificate<br>• TAFE diploma<br>• University undergraduate degree<br>• University postgraduate degree
Q9	Are you a parent of a child who is currently under the age of 18?	• Yes<br>• No
Q10	Are you an early childhood educator, primary school or high school teacher?	• Yes<br>• No
	**Awareness of myopia**	
Q11	Select the option that is **most** true for you regarding the term myopia:	• I have heard the name and know a lot about the symptoms<br>• I have heard the name and know a little about the symptoms<br>• I have heard the name but don't know the symptoms<br>• I have never heard of it
Q12	Have you been diagnosed with myopia or do you think you may have myopia?	• Yes<br>• Unsure<br>• No
Q13	Select the option you believe to be correct	
Q13.1	Myopia is also known as ...	• Short-sightedness<br>• Long-sightedness

<table>
<tr><td>Q13.2</td><td>Myopia is a condition where ...</td><td>Near objects are hard to see clearly
Distant objects are hard to see clearly</td></tr>
<tr><td>Q13.3</td><td>Myopia is becoming more ...</td><td>Common
Rare</td></tr>
<tr><td>Q13.4</td><td>Poor eye muscle control is ...</td><td>Not related to myopia
Involved with myopia</td></tr>
<tr><td>Q13.5</td><td>Myopia is a condition that often involves accelerated eye growth</td><td>False
True</td></tr>
<tr><td>Q14</td><td colspan="2">Please indicate the extent to which you agree or disagree with the following statements:</td></tr>
<tr><td>Q14.1</td><td>Too much time spent reading increases the risk of developing myopia</td><td rowspan="6">5 Strongly Agree
4 Agree
3 Neutral
2 Disagree
1 Strongly Disagree</td></tr>
<tr><td>Q14.2</td><td>Too much screen time can cause myopia to develop or worsen it</td></tr>
<tr><td>Q14.3</td><td>It is better not to wear eyeglasses/visual correction, even when you have myopia, since it may very likely make your vision worse</td></tr>
<tr><td>Q14.4</td><td>Corrective lens power should be less than your real power since the full correction lenses may accelerate myopia progression</td></tr>
<tr><td>Q14.5</td><td>Refractive laser eye surgery will fix myopia and reduce the risk of other eye diseases it may be associated with</td></tr>
<tr><td>Q14.6</td><td>There are eye exercises to cure or reverse myopia</td></tr>
</table>

Q14.7	Having myopia poses a risk for vision-threatening complications later in life	
Q14.8	High myopia is associated with increased risk of irreversible blindness	
Q14.9	Myopia is a public health concern with socio-economic problems	
Q14.10	Early intervention is key to minimizing the amount of reduced vision caused by myopia	
Q14.11	Myopia can cause poor academic performance and behavioural issues at school	
Q14.12	School vision screenings are sufficient in detecting eye problems in children	
Q15	What do you believe is the **<u>minimum</u>** distance it is safe to hold device screens from the eyes?	• 10cm<br>• 20cm<br>• 30cm<br>• 40cm
Q16	From 1 to 10 rate your level of concern about **<u>your</u>** myopia, and about the progressive increase of myopia, or the possibility of having or developing myopia:	Participants could drag a scale from 1 to 10.<br>1 = Not at all 10 = Extremely Concerned
Q17	Indicate the reason(s) you are concerned about **<u>your</u>** myopia, developing myopia, or progressing myopia (you can choose several reasons):	• Aesthetics or personal image<br>• Economical cost for spectacle or contact lenses

		• Dependence on spectacles or contact lenses<br>• Ocular health<br>• Other (indicate if possible):
Q18	Select the option(s) that is true for you.	
	I have heard of or know the following possible consequence of having high myopia:	• I have never heard that having myopia can lead to visual health consequences<br>• Cataract development<br>• Retinal detachment<br>• Glaucoma development<br>• Other (please specify):
	Awareness of Myopia Interventions	
Q19	Did you know that treatments now exist that could slow down the progression of myopia or help to stop it from getting worse?	• Yes<br>• No
Q20	Do you know of any of these myopia control methods to control the progression of myopia? Please indicate all you are familiar with below:	• Medicated eyedrops, Atropine<br>• Soft contact lenses for myopia control, Misight<br>• Wearing rigid (hard) contact lenses at night, orthokeratology or Ortho-K<br>• Spectacles to control myopia, Miyosmart, Stellest<br>• Progressive addition spectacles<br>• Repeated low-level red light (RLRL) therapy

		• I did not know any method of myopia control
	**Parents and Myopia Section**	
Q21	For the following questions, please answer considering your child who is below 18 years old. If you have more than one child, consider the child for whom myopia appeared first, or the oldest child under 18 if no myopia is present.	
Q22	What is the child's age?	Parents could select from the ages of 1 to 17.
Q23	Has your child had their eyes tested at a school vision screening?	• Yes<br>• No<br>• Unsure
Q24	What age did this child have their first eye test with an optometrist or ophthalmologist?	• 3 years or younger<br>• 4-8 years<br>• 9-11 years<br>• 12-17 years<br>• Not had an eye test
Q25	What prompted you to schedule the child's first eye test? (select all that apply)	• They made complaints about their symptoms.<br>• You observed certain behaviours.<br>• Raised awareness from campaigns about children eye exams Eg. social media, TV, marketing<br>• Eye related injury<br>• There is family history of myopia

		• There is family history of eye conditions and/or vision loss<br>• It was recommended by teachers/school<br>• It was recommended following a vision screening test<br>• It was suggested by a family member/friend<br>• No Particular reason other than routine exam
Q26	The child's mother has: (multiple selections allowed)	
Q27	The child's father has: (multiple selections allowed)	• Myopia<br>• Hyperopia<br>• Astigmatism<br>• Presbyopia<br>• None of them<br>• I don't know
Q28	Does your child have myopia?	• No<br>• Yes<br>• Unsure
Q30	How many dioptres(D) of myopia does the child have (what is their prescription?	• Between 0.25D and 0.75D<br>• Between 1D and 1.75D<br>• Between 2D and 3.75D<br>• More than 4D

		• Unsure
Q31	During the last year, the child's myopia has increased by:	• It has not increased<br>• 0.25D<br>• 0.50D<br>• 0.75D<br>• 1D or more<br>• I don't know<br>• I don't know if it has increased
Q32	You would say that your child's myopia is progressing:	• Very slowly<br>• Slowly<br>• Moderately<br>• Fast<br>• Very Fast<br>• Unsure
Q33	When your child was diagnosed with myopia did the optometrist discuss that this is a condition that will commonly progress?	• Yes<br>• Unsure<br>• No
Q34	When your child was diagnosed with myopia did the optometrist discuss there are special myopia management options that my help slow its progression?	• Yes<br>• Unsure

		• No
Q35	From 1 to 10 rate your level of concern about **<u>your child's</u>** myopia, and about the progressive increase of myopia, or the risk you child may develop myopia:	Participants could drag a scale from 1 to 10.<br>1 = Not at all<br>10 = Extremely Concerned
Q36	Indicate the reason(s) you are concerned that **<u>your child</u>** has myopia (or may develop myopia), and that it is progressing (you can choose several reasons).	• Aesthetics or personal image<br>• Economical cost for spectacle or contact lenses<br>• Dependence on spectacles or contact lenses<br>• Ocular health<br>• Other (indicate if possible):
Q37	If you are concerned your child may have myopia, rank, in order from first to last, where you would go for more information.	• Internet/online<br>• Local doctor/GP<br>• Optometrist<br>• Ophthalmologist<br>• Child's teacher<br>• Family/friends
Q38	While at home, how many hours a day does your child spend looking at digital devices?	Participant to fill in
Q39	Please indicate the extent to which you agree or disagree with the following statements	

Q39.1	I am concerned about the amount of time my children spend looking at screens	• 5 Strongly Agree<br>• 4 Agree<br>• 3 Neutral<br>• 2 Disagree<br>• 1 Strongly Disagree
Q39.2	I am concerned that my children's screen time will damage their eyesight	
Q40	In the past year, how often have you done the following:	
Q40.1	Remind my child what the correct distance between their eyes and the book/near electronic device should be	• 5 Strongly Agree<br>• 4 Agree<br>• 3 Neutral<br>• 2 Disagree<br>• 1 Strongly Disagree
Q40.2	When the child is reading a book, or using a smart phone, tablet, computer or watching TV, interrupt them every 30-40 minutes and let them look far away and rest for a few minutes	
Q40.3	Encourage outdoor play time every day	
Q40.4	Adjust my child's lighting conditions when they are doing near work or reading	
	**Teachers and Myopia Section**	
Q41	Considering the most recent place you have taught at:<br>Does your school have measures in place for potentially detecting and informing parents of myopia?	• No<br>• Unsure

		• Yes
Q42	Are you aware of potential resources to improve your understanding of myopia?	• No<br>• Unsure<br>• Yes
Q43	Which of these signs, if any, would prompt you to consider whether a student has myopia? (select all that apply)	• Squinting, turning or tilting their head to see<br>• Eye rubbing<br>• Complaining that they cannot see the whiteboard<br>• Complaints of headaches<br>• Holding books very close to their face<br>• Sitting very close to the TV/projector<br>• Poor grades<br>• Covering one eye to read<br>• Behavioral problems<br>• Using a finger to follow along while reading<br>• None of the above
Q44	Please indicate the extent to which you agree or disagree with the following statements	
Q44.1	I feel confident in my understanding of myopia to detect potential signs of it in my classroom	• 5 Strongly Agree

Q44.2	I am confident in recommending parents take their children for an eye exam	• 4 Agree<br>• 3 Neutral<br>• 2 Disagree<br>• 1 Strongly Disagree<br>• N/A
Q44.3	If I have recommended a child have an eye exam, I always follow up verbally	
Q44.4	If I have recommended a child have an eye exam, I always request a written report back	
Q44.5	I believe it is within my scope and duty to understand and detect conditions such as myopia in my students	

Supplementary Table. 2 *Demographics of survey participants compared to the Australian population, based on the Australian Bureau of Statistics (ABS) and Australian Institute of Health and Welfare (AIHW) data set*

	Variable	Study Sample, n (%)	ABS, n (%)
Gender*	Female	739 (49.9)	12,877,635 (50.7)
	Male	739 (49.9)	12,545,154 (49.3)
	Other	4 (0.3)	
Age (years)[+]	0-19	14 (0.9)^	6,456,029 (23.7)
	20-49	753 (50.8)	11,280,381 (41.5)
	50-79	680 (45.9)	8,264,388 (30.4)
	80+	35 (2.4)	1,193,571 (4.4)
State/Territory[•]	NSW	469 (31.6)	8,511,151 (31.2)
	VIC	372 (25.1)	7,012,962 (25.7)
	QLD	304 (20.5)	5,608,666 (20.5)
	WA	157 (10.6)	2,981,752 (10.9)
	SA	110 (7.4)	1,882,722 (6.9)
	TAS	31 (2.1)	575,559 (2.1)
	ACT	26 (1.8)	475,644 (1.7)
	NT	13 (0.9)	255,559 (0.9)
Ethnicity	Oceanian	839 (56.6)	N/A
	Northwest European	294 (19.8)	
	Southern and Eastern European	105 (7.1)	
	Southeast Asian	74 (5.0)	
	Northeast Asian	71 (4.8)	
	Southern and Central Asian	49 (3.3)	
	North African and Middle Eastern	17 (1.1)	
	Peoples of the Americas	16 (1.1)	
	Sub-Saharan African	7 (0.5)	
	Did not say	10 (0.7)	
Level of highest educational attainment[§]	Completed high school (Year 12)	277 (18.7)	3,104,116 (14.9)
	Vocational education and training	422 (28.5)	5,300,230 (25.6)
	University bachelor's degree or higher	690 (46.5)	5,464,631 (26.3)
	Other[Δ]	92 (6.2)	6,912,429 (33.2)

* National representative data derived from: www.abs.gov.au/statistics/people/people-and-communities/snapshot-australia/2021

[+] National representative data derived from: www.aihw.gov.au/reports/Australias-health/profile-of-Australias-population

^ Study age limit was ≥18 years accounting for the low proportion in comparison the national data

• National representative data derived from: www.dataexplorer.abs.gov.au - Quarterly Population Estimates (ERP), by State/Territory, Sex and Age (2024-Q3)

[§] National representative data derived from: https://www.abs.gov.au/census/find-census-data/quickstats/2021/AUS

[Δ] Includes individuals who indicated they had completed some high school years for our study, meanwhile for the national data set represents schooling years 11 and below, responses that were inadequately described, those without educational attainment, as well as those who did not state a response
